# Comparative genomics analyses revealed two virulent *Listeria monocytogenes* strains isolated from ready-to-eat food

**DOI:** 10.1186/s13099-016-0147-8

**Published:** 2016-12-12

**Authors:** Shu Yong Lim, Kien-Pong Yap, Kwai Lin Thong

**Affiliations:** Faculty of Science, Institute of Biological Sciences, University of Malaya, 50603 Kuala Lumpur, Malaysia

**Keywords:** *Listeria monocytogenes*, Comparative genomics, Multidrug resistant, Ready-to-eat food, LIPI-1

## Abstract

**Background:**

*Listeria monocytogenes* is an important foodborne pathogen that causes considerable morbidity in humans with high mortality rates. In this study, we have sequenced the genomes and performed comparative genomics analyses on two strains, LM115 and LM41, isolated from ready-to-eat food in Malaysia.

**Results:**

The genome size of LM115 and LM41 was 2,959,041 and 2,963,111 bp, respectively. These two strains shared approximately 90% homologous genes. Comparative genomics and phylogenomic analyses revealed that LM115 and LM41 were more closely related to the reference strains F2365 and EGD-e, respectively. Our virulence profiling indicated a total of 31 virulence genes shared by both analysed strains. These shared genes included those that encode for internalins and *L. monocytogenes* pathogenicity island 1 (LIPI-1). Both the Malaysian *L. monocytogenes* strains also harboured several genes associated with stress tolerance to counter the adverse conditions. Seven antibiotic and efflux pump related genes which may confer resistance against lincomycin, erythromycin, fosfomycin, quinolone, tetracycline, and penicillin, and macrolides were identified in the genomes of both strains.

**Conclusions:**

Whole genome sequencing and comparative genomics analyses revealed two virulent *L. monocytogenes* strains isolated from ready-to-eat foods in Malaysia. The identification of strains with pathogenic, persistent, and antibiotic resistant potentials from minimally processed food warrant close attention from both healthcare and food industry.

## Background

*Listeria monocytogenes* (*L. monocytogenes*) is a Gram-positive, motile, rod-shaped bacterium that is ubiquitous in nature. It is an emerging foodborne pathogen and causes human listeriosis which can be a life-threatening illness particularly in elderly, pregnant women, new-borns, and immunocompromised patients [1]. Listeriosis has been detected in many geographical regions, particularly in USA and Europe [1]. Although the occurrence of *L monocytogenes* in foods has been detected in Malaysia, cases of listeriosis are rarely reported [2, 3].

Human listeriosis has been associated with the consumption of contaminated raw, processed, and ready-to-eat foods (RTE) [3]. Since *L. monocytogenes* is able to survive in a wide range of adverse conditions such as low temperature (2–4 °C), low pH, and low water content [4], it may outcompete other microorganisms in acidic and refrigerated food, as well as food that are preserved through salting, sugaring and drying. Furthermore, the increasing demand for fresh and minimally processed foods by consumers has increased the risk of listerosis as such foods contain low levels of preservative which can inhibit the growth of *L. monocytogenes* [5].

Serotyping based on the somatic (O) and flagellar (H) antigens has identified 13 serotypes (1/2a, 1/2b, 1/2c, 3a, 3b, 3c, 4a, 4ab, 4b, 4c, 4d, 4e, and 7) in *L. monocytogenes* [6]. The majority of the human listeriosis cases were associated with serotype 4b, 1/2a, 1/2b, and 1/2c [7]. The pathogenicity of these serotypes is mainly attributed to the presence of the *Listeria* pathogenicity island 1 (LIPI-1) which harbours several important virulence genes (*prfA*, *plcA*, *hly*, *mpl*, *actA*, *plcB*). This array of genes promotes cytosolic proliferation as well as intra- and intercellular movement, which are the key processes in the intracellular parasitic life cycle of *L. monocytogenes* [8]. Besides, *L. monocytogenes* also carries *inlA* and *inlB* gene which encode for internalins that help in adherence to and invasion of host cells [9].

*Listeria monocytogenes* is naturally susceptible to a wide range of clinically-relevant antibiotics except for quinolone, fosfomycin and cephalosporins [10]. However, resistance to single or multiple antibiotics has increasingly been reported for food strains [3, 11]. The occurrence of resistant strains might be a consequence of food contamination by the food handlers or from the contaminated food processing plants. Apart from that, the use of antibiotics in livestock as growth promoter or for disease treatment and prevention may act as a selective pressure for emerging resistant strains which may be zoonotically transferred to humans via food consumption [12]. Given the severity of listeriosis, the emergence of antibiotic resistant *L. monocytogenes* poses a major health concern in both food safety and public health.

The availability of complete genome sequence of *L. monocytogenes* allows comparative genomics analyses to be performed, which shed light on the genetic basis underlying the virulence and adaptability of this foodborne pathogen. New genomic data is needed to extend our understanding on the pathogenicity of this organism. This new genomic information may help in the development of new control method through identification and discovery of new virulence-associated genes. In this study, we sequenced and analysed two *L. monocytogenes* strains isolated from RTE food in Malaysia to elucidate their virulence potential. Genomic comparison was also performed between the studied strains and three other reference strains to gain insights into the evolutionary relationships of these bacteria.

## Methods

### Bacteria strains and genomic DNA extraction

LM115 and LM41 were isolated from fried fish and salad, respectively, that were purchased from a Malaysian street-side hawker stall in 2011 as previously described [2]. The strains were cultivated in Trytic soy medium (Oxoid, Basingstoke, UK) and preserved at −80 °C in 50% glycerol. The genomic DNA was extracted from a pure culture using DNeasy Blood & Tissue kit (Qiagen, Hilden, Germany) according to the manufacturer’s instruction.

### Whole genome sequencing, assembly, and annotation

Whole genome sequencing of the *L. monocytogenes* strains was performed on an Illumina HiSeq 2000 platform. The generated sequence reads were trimmed, quality-checked, and assembled *de novo* using CLC Genomics Workbench 5.1 (CLC Bio, Denmark) as previously described [13]. A total of 28 and 11 contigs with the coverage of 98× and 101× were generated for LM115 and LM41, respectively. These contigs were mapped and reordered against *L. monocytogenes* EGD-e (1/2a) using Mauve [14]. Assembled sequence was then submitted to the Rapid Annotation using Subsystem Technology (RAST) server [15] for annotation. The number of rRNA was predicted using RNAmmer 1.2 server [16] whereas the numbers of tRNA and tmRNA were gleaned through ARAGORN [17].

### Comparative genomics and phylogenomic analysis

Comparative genomics analysis was performed among the two Malaysian *L. monocytogenes* strains, *L. monocytogenes* strain EGD-e (1/2a), F2365 (4b), and *L. innocua* CLIP 11262 (6a) by identifying and comparing the homologous and orthologous genes of these five strains using Pan-Genomes Analysis pipeline (PGAP) [18]. BLAST ring image generator (BRIG) was also used for the genomes comparison by performing BLASTn (70 and 50% upper and lower identity threshold, respectively), using strain EGD-e as the reference. Cluster of orthologous group (COG) analysis was performed by assigning all representative protein sequences from each orthologous protein cluster based on local BLASTp against COG database. To study the phylogenetic relationship of LM115 and LM41, the genomes of 15 other *Listeria* strains were also included for comparison. The strains and GenBank accession numbers are as follows: EGD-e (NC_003210), F6854 (AADQ01000001), H7858 (AADR01000001), HCC23 (NC_011660), SLCC2376 (NC_018590), F2365 (NC_002973), LM201 (AYPT01000001), Clip80459 (NC_012488), SLCC2540 (NC_018586), S1_4 (JWHI01000001), SLCC7179 (NC_018593), LM3136 (NZ_CP013723), Scott A (CM001159), FSL N3-165 (AARQ02000001), Clip11262 (NC_003212). All these strains belong to the pathogenic serotypes (4b, 1/2a, 1/2b, and 1/2c) except for HCC23 (4b), SLCC2376 (4c), and Clip11262 (6a) which serve as an outgroup. A single-nucleotide polymorphism (SNP) based phylogeny tree, using strain EGD-e as the reference genome, was inferred by CSI Phylogeny 1.4. [19] using the default parameters. Briefly, SNPs were called by mapping the genomes of the studied strains to that of the reference. Site validation of the called SNPs was performed and a phylogeny tree was inferred based on the concatenated alignment of the quality-checked SNPs. The phylogeny tree inferred was viewed using FigTree software (http://tree.bio.ed.ac.uk/software/figtree/).

### Virulence factors and antimicrobial resistance genes identification

Virulence genes were predicted by performing a BLAST search of LM115 and LM41 genomes against the Virulence Factors of Pathogenic Bacteria database (VFDB) [20]. For antimicrobial resistance genes detection, the whole genome sequences of LM115 and LM41 were uploaded to the Resistance Gene Identifier (RID) of the Comprehensive Antibiotic Resistance Database (CARD) [21]. The predicted genes were then validated by performing BLASTp against both the non-redundant (nr) and Swiss-Prot database with 60% coverage and 60% sequence identity as the threshold. If results of the two databases conflicted, a priority order of nr, Swiss-Prot was followed.

## Quality assurance

Standard biochemical tests (Gram staining, catalase, oxidase, urea, SIM, TSI, and MR-VP) and species-specific PCR were used to confirm the identity of both *L. monocytogenes* strains LM115 and LM41 as previously described [2]. Genomic DNA was extracted from a single colony of the pure bacterial culture. Potential contamination of the genomic library by foreign DNA was assessed using the CLC Genomics Workbench 5.1 (CLC Bio, Denmark) as previously described [13].

## Results and discussion

### General genome features

The predicted genome sizes of LM115 and LM41 are 2,959,041 and 2,963,111 bp, respectively. The G + C contents of the two genomes are approximately 38%. The number of tRNA is 51 and 60 for LM115 and LM41, respectively. Both strains carry three rRNA and one tmRNA. A total of 2913 and 2951 coding sequences (CDS) were predicted for LM115 and LM41, respectively. The genome features of the two strains are summarized in Table 1.

**Table 1:** General genome features of *Listeria monocytogenes*, LM115 and LM41

	LM115	LM41
Chromosome size (bp)	2,959,041	2,963,111
GC (%)	37.85	37.82
Number of CDS	2913	2951
Number of rRNA	3	3
Number of tRNA	51	60
Number of tmRNA	1	1

### Comparative genomics and phylogenomic analysis

**Fig. 1:**
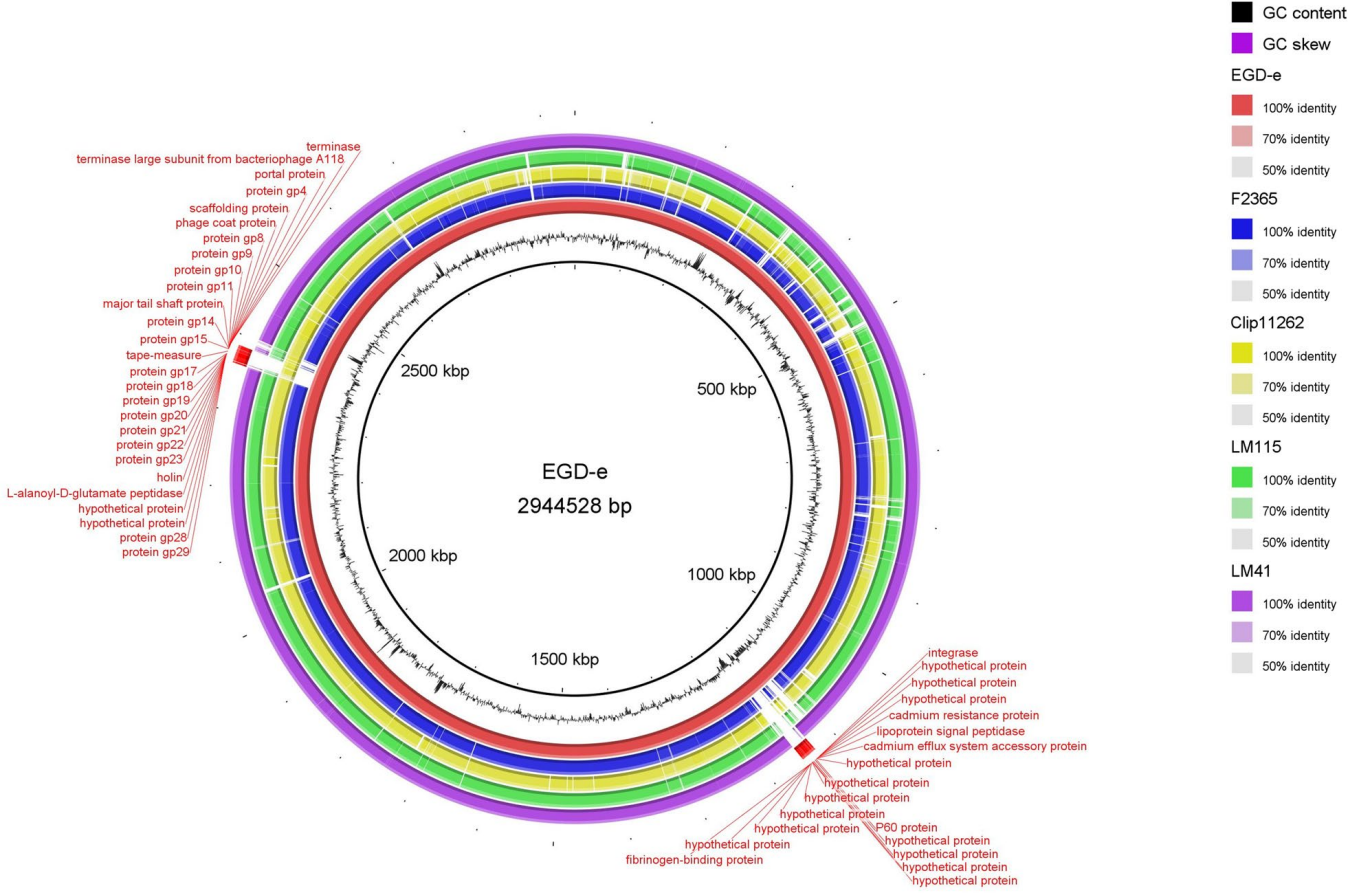
Circular genomic map and genome comparison of *L. monocytogenes* LM115, LM41, EGD-e, F2365, and *L innocua* Clip11262. The *inner ring* shows coordinate in scale and the total genome size of the reference sequence, EGD-e. The *black histogram bar* represents G + C content whereas the *purple*-*green histogram bar* represents G + C skew. *Coloured arches* representing orthologous regions of each genome in respect to EGD-e (*red arch*) and are shown in the following order (*inside to outside*): EGD-e (*red*), F2365 (*blue*), Clip11262 (*yellow*), LM115 (*green*), LM41 (*purple*)

Whole genome comparison of the two Malaysian *L. monocytogenes* strains with *L. monocytogenes* EGD-e, F2365, and *L. innocua* Clip11262 revealed a total of 2497 shared ORFs, which accounted for approximately 82% of the total ORFs present in each of the studied strains. LM41 was genetically more similar to EGD-e, a derivative of an animal isolate EGD that was used in cell-mediated immunity studies [22]. This genetic similarity was depicted in the circular genomic map of genomes comparison (Fig. 1) where LM41 showed high genome identity (>70% nucleotide identity) to EGD-e, except for two major regions in EGD-e, ranging from approximately 1132–1152 kb and 2362–2385 kb. These regions carried genes that encode for various proteins, including hypothetical proteins, cadmium resistance protein, and phage-related proteins. In contrast, LM115 showed less genomic similarity with EGD-e but was more closely related to F2365, a cheese isolate from a Californian outbreak in 1985 [23] (Fig. 1).

**Fig. 2:**
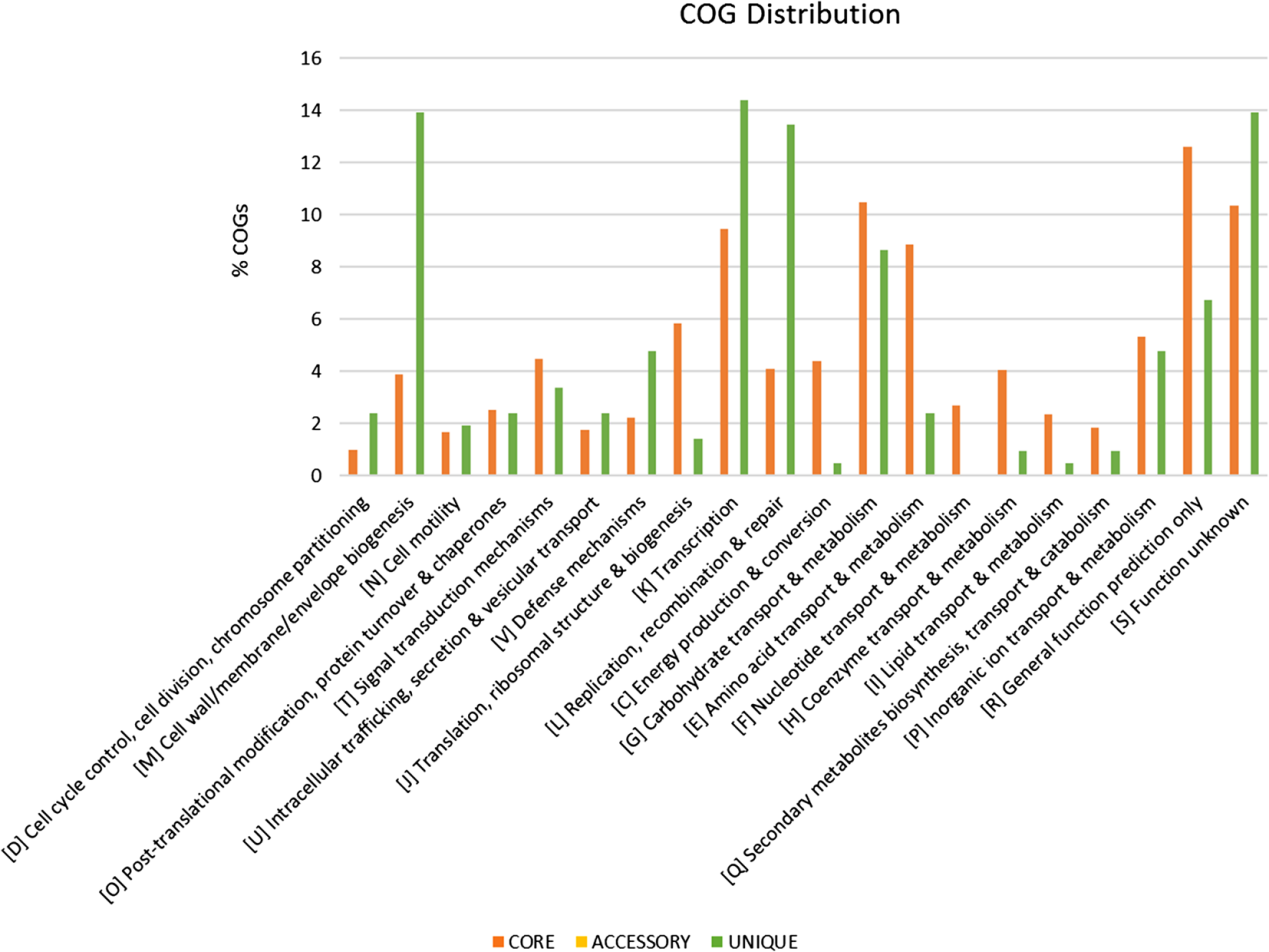
COG distribution of core and unique genes of LM115 and LM41. The *vertical axis* represents the percentage of genes (core, orthologous and singleton) distributed to specific COG class between LM115 and LM41 whereas the *horizontal axis* represents different functional classes of COG

Pairwise comparison showed that LM115 and LM41 shared approximately 90% of their total ORFs. The core and unique genes of LM115 and LM41 were further analysed according to various classes of Cluster of Orthologous Groups (COGs) (Fig. 2). Our results showed that genes from COG class J (Translation, ribosomal structure biogenesis), class C (Energy production conversion), class E (Amino acid transport metabolism), class F (Nucleotide transport metabolism), and class H (Coenzyme transport metabolism) were abundant in the core genome. On the other hand, the unique genes were mostly associated with class M (Cell wall/membrane/envelope biogenesis), class V (Defence mechanism), and class L (Replication, recombination conversion). Detailed genome analysis showed that LM115 and LM41 carried a total of 95 and 116 strain-specific genes, respectively. Other than genes related to the mentioned COG classes (M, V, and L), most of the unique genes encode for hypothetical proteins. To note, these strain-specific hypothetical proteins might carry functions relevant to specific adaptive or fitness advantages, despite the fact that their functions remain uncharacterized.

**Fig. 3:**
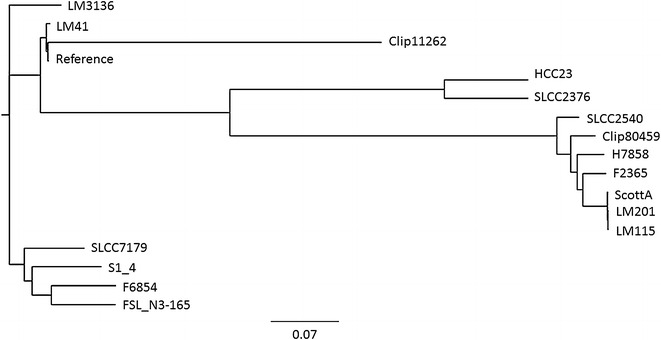
SNP-based phylogeny tree of 17 *Listeria* strains. The phylogeny tree was generated using CSI Phylogeny 1.4 [19]. Single nucleotide polymorphisms (SNPs) of each strain were called by mapping the genome sequence to that of the reference. The quality-checked SNPs were then concatenated and used to infer a maximum-likelihood tree. The “Reference” refers to the Reference strain *L. monocytogenes* EGD-e

Our SNP-based phylogenomic analysis showed that LM41 and LM115 were closely related to the reference strain EGD-e and F2365 (Fig. 3), respectively, consistent with the findings of our comparative genomics discussed earlier (Fig. 1). LM115 was also shown to be closely related to two serotype 4b strains, LM201 and Scott A. LM201 is isolated from foodstuffs in China whereas Scott A is a clinical isolate from the Massachusetts listeriosis outbreak in 1983 [24, 25]. The phylogenomic tree also revealed the separation of LM115 and LM41 into two different clades. Since SNPs were used to infer the phylogeny relationship of these strains, this observation indicated a possibly high genetic variation in the genomes of LM115 and LM41.

### Virulence genes profiling

Several virulence genes found in *Listeria* spp. were shared between LM115 and LM41. These included the *Listeria* pathogenicity island (LIPI-1) and several internalins. The LIPI-1 plays a major role in the pathogenicity of *L. monocytogenes* and consists of six genes that are important for phagosomal escape (*hly*, *plcA*, *plcB*, *mpl*), motility and cell-to-cell spread (*act*), and gene regulation (*prfA*) [8]. Six internalins genes (*inlA, inlB, inlC, inlK, inlF, inlJ*) were identified in both LM115 and LM41. These internalin genes are involved in invasion (*inlA*, *inlB*), adherence (*inlF*, *inlJ*), cell-to-cell spread (*inlC*), and autophagy evasion (*inlK*) [9, 26, 27]. Other virulence factors that were annotated in the genomes of both LM115 and LM41were bile salt hydrolase (*bsh*) which provides resistance to the acute toxicity of bile salt in the host intestine and autolysis amidase (*ami*) which plays a role in host cells adhesion [28, 29]. All the virulence genes identified in both LM115 and LM41 were also present in the pathogenic reference strains EGD-e and F2365.

### Stress tolerance

*Listeria monocytogenes* can encounter various stresses due to the different food processing methods such as heating, chilling, and sugaring. The ability of this pathogen to adapt to and overcome these stresses is contributed by their stress tolerance proteins. A number of genes encoding stress response proteins were identified in LM115 and LM41 (Table 2). Both strains carried the glutamate decarboxylase (GAD) operon and arginine deiminase (ADI) operon that are involved in acid tolerance. The GAD system increases the pH of cytoplasm by utilizing intracellular proton during the conversion of glutamate to ϒ-aminobutyrate (GABA) [30]. The ADI system, on the other hand, alleviates the acidity of cytoplasm by combining intracellular proton with the system’s by-product (NH_3_) to release ammonium ion (NH_4_^+^) [31]. Both these systems may provide competitive advantage to *L. monocytogenes* to survive in food with low pH which can usually limit bacterial growth. In fact, the role of the GAD system in acid tolerance had been demonstrated in acidified skim milk [32] and cheese [33]. Besides, LM115 and LM41 also harboured several cold and heat shock proteins related genes which protect bacteria from cell damage induced by temperature stress [34, 35]. Foods stored in low temperature or processed with high heat, such as frozen burger patties and fried chickens, had been reported to contaminated with *L. monocytogenes* in Malaysia [2, 36]. Moreover, BetL, Gbu, and OpuC transport systems which play a major role in *L. monocytogenes* osmotic stress response were also annotated in the genomes of LM115 and LM41. These three systems are involved in the uptake of betaine and carnitine that balance the intracellular and extracellular osmotic stress [37], allowing *L. monocytogenes* to survive in food preserved in low water content. Gene encoding the sigma-B regulator protein (SigB) which regulates various stress responses such as osmotic and temperature stress was also identified in LM115 and LM41.

**Table 2:** Stress response genes identified in *Listeria monocytogenes* LM115 and LM41

Environmental stress	Gene	LM115	LM41
General			
Sigma-B regulator protein	*sigB*	+	+
Acid			
Glutamate decarboxylase system	*gadD1*	+	+
	*gadD2*	+	+
	*gadD3*	+	+
	*gadT1*	+	+
	*gadT2*	+	+
Arginine and agmatine systems	*lmo0036*	+	+
	*lmo0037*	+	+
	*lmo0038*	+	+
	*lmo0039*	+	+
	*lmo0040*	+	+
	*lmo0041*	+	+
	*lmo0042*	+	+
	*lmo0043*	+	+
Temperature			
Cold shock protein	*cspA*	+	+
	*cspB*	+	+
	*cspD*	+	+
Heat shock protein	*dnaK*	+	+
	*dnaJ*	+	+
	*hrcA*	+	+
	*grpE*	+	+
Water			
Osmotolerance proteins	*gbuA*	+	+
	*gbuB*	+	+
	*gbuC*	+	+
	*opuCA*	+	+
	*opuCB*	+	+
	*opuCC*	+	+
	*opuCD*	+	+
	*opuD*	+	+
	*betL*	+	+

+ Represents the presence of stress response gene

### Antibiotic resistance determinants

Both LM115 and LM41 carried similar antibiotic resistance related genes in their genomes. The *tetA* gene which is related to tetracycline resistance was found in both strains. Although an association of *tetM* to tetracycline resistance was more commonly reported, *tetA* had also been identified in strains isolated from fish samples [38, 39]. Additionally, LM115 and LM41 also harboured *mecC* gene which could confer resistance to beta-lactam drugs. Beta lactam antibiotics such as ampicillin and penicillin, in combination with aminoglycosides, remain the primary therapeutic option for human listeriosis [40]. Resistance to beta lactam drugs could challenge the current treatment option in effectively treating the disease. Apart from that, genes encoding for lincomycin resistance protein (*lmrB*), fosfomycin resistance protein (*fosX*), and erythromycin resistance ATP-binding protein (*msrA*) were also identified in both strains. Furthermore, two efflux pump-related genes, *lde* and *mdrL*, which confer resistance to quinolone and macrolides, respectively, were also identified in the two genomes.

A few recent reports have documented the isolation of resistant *L. monocytogenes* strains against one or more antibiotics in Malaysia [3, 9]. The isolation of resistant strains from food is an important health risk as these strains could be transmitted to humans via food contamination. The identification of multiple antibiotic resistance genes in LM115 and LM41 further reiterates the importance of food practice to prevent the dissemination of this pathogen.

## Conclusions

Our comparative genomics analyses identified approximately 90% homologous genes between LM115 and LM41. Both LM115 and LM41 showed a close phylogenetic relationship with the pathogenic reference strains F2365 and EGD-e, respectively. Based on our initial genomic analysis, several virulence genes such as those encode for LIPI-1 and internalins were shared between the two strains. Both LM115 and LM41 harboured several stress tolerance genes which may help them to survive through various stresses imposed by different food processes. Additionally, a number of antibiotic resistance genes were also found in the two genomes. The occurrence of virulent and antibiotic resistant *L. monocytogenes* strains with significant stress tolerance in RTE food poses a great concern for food safety. Functional genomic studies are required to study the association of these genes to the persistence and pathogenicity of these strains.

## Abbreviations

RTE: ready-to-eat foods
LIPI-1: *Listeria* pathogenicity island 1
RAST: rapid annotation using subsystem technology
PGAP: pan-genomes analysis pipeline
SNP: single-nucleotide polymorphism
VFDB: virulence factors of pathogenic bacteria database
RID: resistance gene identifier
CARD: comprehensive antibiotic resistance database
CDS: protein coding sequences
PCR: polymerase chain reaction
SIM: sulfide-indol-motality
TSI: tripe sugar iron
MR-VP: methyl red-Voges proskauer
GAD: glutamate decarboxylase
ADI: arginine deiminase
COG: cluster of orthologous group
BRIG: BLAST ring image generator

## References

[1] Lomonaco S, Nucera D, Filipello V (2015). The evolution and epidemiology of *Listeria monocytogenes* in Europe and the United States. Infect Genet Evol.

[2] Jamali H, Chai LC, Thong KL (2013). Detection and isolation of *Listeria* spp. and *Listeria monocytogenes* in ready-to-eat foods with various selective culture media. Food Control.

[3] Marian MN, Sharifah Aminah SM, Zuraini MI, Son R, Maimunah M, Lee HY (2012). MPN-PCR detection and antimicrobial resistance of *Listeria monocytogenes* isolated from raw and ready-to-eat foods in Malaysia. Food Control.

[4] Gandhi M, Chikindas ML (2007). Listeria: a foodborne pathogen that knows how to survive. Int J Food Microbiol.

[5] McCollum JT, Cronquist AB, Silk BJ, Jackson KA, O’Connor KA, Cosgrove S (2013). Multistate outbreak of listeriosis associated with cantaloupe. N Engl J Med.

[6] Seeliger H, Höhne K (1979). Serotyping of *Listeria monocytogenes* and related species. Methods Microbiol..

[7] Swaminathan B, Gerner-Smidt P (2007). The epidemiology of human listeriosis. Microbes Infect.

[8] Vazquez-Boland JA, Dominguez-Bernal G, Gonzalez-Zorn B, Kreft J, Goebel W (2001). Pathogenicity islands and virulence evolution in Listeria. Microbes Infect.

[9] Bierne H, Sabet C, Personnic N, Cossart P (2007). Internalins: a complex family of leucine-rich repeat-containing proteins in *Listeria monocytogenes*. Microbes Infect.

[10] Poros-Gluchowska J, Markiewicz Z (2003). Antimicrobial resistance of *Listeria monocytogenes*. Acta Microbiol Pol.

[11] Jamali H, Thong KL (2014). Genotypic characterization and antimicrobial resistance of *Listeria monocytogenes* from ready-to-eat foods. Food Control.

[12] Economou V, Gousia P (2015). Agriculture and food animals as a source of antimicrobial-resistant bacteria. Infect Drug Resist..

[13] Yap KP, Ho WS, Gan HM, Chai LC, Thong KL (2016). Global MLST of *Salmonella* Typhi revisited in post-genomic era: genetic conservation, population structure, and comparative genomics of rare sequence types. Front Microbiol..

[14] Darling ACE, Mau B, Blattner FR, Perna NT (2004). Mauve: multiple alignment of conserved genomic sequence with rearrangements. Genome Res.

[15] Aziz RK, Bartels D, Best AA, DeJongh M, Disz T, Edwards RA (2008). The RAST server: rapid annotations using subsystems technology. BMC Genom.

[16] Lagesen K, Hallin P, Rødland EA, Stærfeldt HH, Rognes T, Ussery DW (2007). RNAmmer: consistent and rapid annotation of ribosomal RNA genes. Nucleic Acids Res.

[17] Laslett D, Canback B (2004). ARAGORN, a program to detect tRNA genes and tmRNA genes in nucleotide sequences. Nucleic Acids Res.

[18] Zhao Y, Wu J, Yang J, Sun S, Xiao J, Yu JPGAP (2012). Pan-genomes analysis pipeline. Bioinformatics.

[19] Kaas RS, Leekitcharoenphon P, Aarestrup FM, Lund O (2014). Solving the problem of comparing whole bacterial genomes across different sequencing platforms. PLoS ONE.

[20] Chen L, Yang J, Yu J, Yao Z, Sun L, Shen Y (2005). VFDB: a reference database for bacterial virulence factors. Nucleic Acids Res.

[21] McArthur AG, Waglechner N, Nizam F, Yan A, Azad MA, Baylay AJ (2013). The comprehensive antibiotic resistance database. Antimicrob Agents Chemother.

[22] Glaser P, Frangeul L, Buchrieser C, Rusniok C, Amend A, Baquero F (2001). Comparative genomics of *Listeria* species. Science.

[23] Mascola L, Lieb L, Fannin SL, Chiu J (1988). Listeriosis: an uncommon opportunistic infection in patients with acquired immunodeficiency syndrome: a report of five cases and a review of the literature. Am J Med.

[24] Wu Y, Zheng J, Wang Y, Li S, Jin H, Li Z (2015). Draft genome sequence of *Listeria monocytogenes* LM201, isolated from foodstuff. Genome Announc..

[25] Briers Y, Klumpp J, Schuppler M, Loessner MJ (2011). Genome sequence of *Listeria monocytogenes* Scott A, a clinical isolate from a food-borne listeriosis outbreak. J Bacteriol.

[26] Dortet L, Mostowy S, Louaka AS, Gouin E, Nahori MA, Wiemer EA, Dussurget O, Cossart P (2011). Recruitment of the major vault protein by InlK: a Listeria monocytogenes strategy to avoid autophagy. PLoS Pathog.

[27] Kirchner M, Higgins DE (2008). Inhibition of ROCK activity allows InlF-mediated invasion and increased virulence of *Listeria monocytogenes*. Mol Microbiol.

[28] Asano K, Kakizaki I, Nakane A (2012). Interaction of *Listeria monocytogenes* autolysin amidase with glycosaminoglycans promotes listerial adhesion to mouse hepatocytes. Biochimie.

[29] Gahan CG, Hill C (2014). *Listeria monocytogenes*: survival and adaptation in the gastrointestinal tract. Front Cell Infect Microbiol..

[30] Cotter PD, Ryan S, Gahan CGM, Hill C (2005). Presence of GadD1 glutamate decarboxylase in selected *Listeria monocytogenes* strains is associated with an ability to grow at low pH. Appl Environ Microbiol.

[31] Chen J, Cheng C, Xia Y, Zhao H, Fang C, Shan Y (2011). Lmo0036, an ornithine and putrescine carbamoyltransferase in *Listeria monocytogenes,* participates in arginine deiminase and agmatine deiminase pathways and mediates acid tolerance. Microbiology.

[32] Cotter PD, O’Reilly K, Hill C (2001). Role of the glutamate decarboxylase acid resistance system in the survival of *Listeria monocytogenes* LO28 in low pH foods. J Food Prot.

[33] Collins B, Cotter PD, Hill C, Ross RP (2011). The impact of nisin on sensitive and resistant mutants of *Listeria monocytogenes* in cottage cheese. J Appl Microbiol.

[34] Melo J, Andrew PW, Faleiro ML (2015). *Listeria monocytogenes* in cheese and the dairy environment remains a food safety challenge: the role of stress responses. Food Res Int.

[35] van der Veen S, Hain T, Wouters JA, Hossain H, de Vos WM, Abee T (2007). The heat-shock response of *Listeria monocytogenes* comprises genes involved in heat shock, cell division, cell wall synthesis, and the SOS response. Microbiology.

[36] Wong WC, Pui CF, Tunung R, Cheah YK, Nakaguchi Y, Nishibuchi M (2012). Prevalence of *Listeria monocytogenes* in frozen burger patties in Malaysia. Int Food Res J..

[37] Sleator RD, Francis GA, O’Beirne D, Gahan CGM, Hill C (2003). Betaine and carnitine uptake systems in *Listeria monocytogenes* affect growth and survival in foods and during infection. J Appl Microbiol.

[38] Jamali H, Paydar M, Ismail S, Looi CY, Wong WF, Radmehr B (2015). Prevalence, antimicrobial susceptibility and virulotyping of *Listeria* species and *Listeria monocytogenes* isolated from open-air fish markets. BMC Microbiol.

[39] Su X, Zhang J, Shi W, Yang X, Li Y, Pan H (2016). Molecular characterization and antimicrobial susceptibility of *Listeria monocytogenes* isolated from foods and humans. Food Control.

[40] Hof H (2004). An update on the medical management of listeriosis. Expert Opin Pharmacother.

